# Spatial and temporal changes in occupancy frequency distribution patterns of freshwater macrophytes in Finland

**DOI:** 10.1002/ece3.7773

**Published:** 2021-06-16

**Authors:** Jukka Suhonen

**Affiliations:** ^1^ Department of Biology University of Turku Turku Finland

**Keywords:** core–satellite species patterns, SOFD patterns, spatial scale, temporal scale, waterbody

## Abstract

A useful method for characterizing biological numerous assemblages at regional scales is the species occupancy frequency distribution (SOFD). An SOFD shows the number or proportion of study sites each species occurred. Species that occur at only a few sites are termed satellite species, while species that occur at many sites are termed core species.

This study is the first to document and assess SOFD patterns in aquatic macrophytes. It characterizes SOFD patterns of freshwater macrophyte assemblages in Finland at two spatial and two temporal scales. For this, I analyzed three published datasets on freshwater macrophyte distributions: two from studies conducted at a local scale and the third from large national surveys. One local study and the national study also included data on temporal variation in species occupancy frequencies.

In the national study, the number of core and satellite species varied slightly between the older and the newer survey, respectively. Among the 113 waterbodies surveyed as part of the national study, the SOFD followed a unimodal satellite pattern. However, for the older dataset (from the 1930s), a bimodal symmetric pattern also fit the SOFD data well. At the local scale, I observed geographical variation in SOFD patterns. The dataset from southern Finland followed a unimodal satellite SOFD pattern; data from central Finland instead displayed a bimodal symmetric SOFD pattern, although they also fit equally well with a bimodal truncated pattern. Moreover, temporal patterns in central Finland seemed to demonstrate a shift from a bimodal symmetric to a bimodal asymmetric SOFD probably.

Geographical variation in the SOFD pattern may be due to variation in the regional species pool. The temporal changes in SOFD pattern may be due to lake eutrophication and anthropogenic disturbance around waterbodies, which may increase number of macrophyte species.

## Graphical abstract

A useful method for characterizing biological numerous assemblages at regional scales is the species occupancy frequency distribution (SOFD). This study is the first to document and assess SOFD patterns in aquatic macrophytes. Geographical variation in the SOFD pattern may be due to variation in the regional species pool and the temporal changes in SOFD pattern may be due to lake eutrophication and anthropogenic disturbance around waterbodies, which may increase number of macrophyte species.

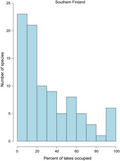

## 1 INTRODUCTION

Ecological studies, characterizing biological assemblages, typically use multiple methods dependent on number of study sites and spatial scales of study. The individual study sites most often used species richness, diversity, abundance of species, and ranked species abundance distributions. In case for small number of different study sites at local and regional scales as a way to look for clusters in graphical ordination and correlations with environmental explanatory variables. Species occupancy frequency distribution patterns (here after SOFD) and ranked species occupancy curves (RSOCs) can be used in comparison for large numbers of sites and a regional spatial scale. It sacrifices information regarding the structure of assemblages at individual sites in order to focus on large‐scale patterns that the other methods cannot address. Both SOFDs and RSOCs can make an important contribution because this pair of methods is uniquely suited to assess assemblage patterns of large numbers of sites at a regional scale (Hui, 2012; Jenkins, 2011; McGeoch & Gaston, 2002; Tokeshi, 1992).

To understand how species richness and SOFD vary in space and time is fundamental to our understanding of the ecology of biological assemblages. The shape of SOFDs varies among assemblages (see reviews by Hui, 2012; Jenkins, 2011; McGeoch & Gaston, 2002; Tokeshi, 1992). Several biological models have been proposed to explain SOFD patterns, but two of them (not mutually exclusive) have received the main support (Jenkins, 2011; McGeoch & Gaston, 2002; Tokeshi, 1992): (a) the dynamic metapopulation model, which is based on local extinction and colonization dynamics (Hanski, 1982b) and (b) the niche‐based model (Brown, 1984) which is based on the idea that assemblages have many rare narrow‐niche specialist species and few common broad‐niche generalist species. In a bimodal core–satellite pattern, many species occur either in only a few sites (satellite species; often rare) or in many sites (core species; often common) (Hanski, 1982b). However, a single‐modality pattern with many satellite species appears to be more common than bimodality; when bimodality has been detected, the most common pattern reported is that the proportion of common species is smaller than that of rare species (Brown, 1984; Hui, 2012; Jenkins, 2011; McGeoch & Gaston, 2002; Tokeshi, 1992). Because the findings of previous studies have not been homogenous, real data on assemblages from different habitats are important for a better understanding of the structure of biological assemblages (Hui, 2012; Tokeshi, 1992).

Describing SOFD patterns in species assemblages yields valuable information for evaluating ecological processes across different spatial (Jenkins, 2011; Jokimäki et al., 2016; Korkeamäki et al., 2018; McGeoch & Gaston, 2002; van Rensburg et al., 2000; Suhonen & Jokimäki, 2019) and temporal scales (Heino, 2008; Jenkins, 2011; McGeoch & Gaston, 2002; Suhonen & Jokimäki, 2019; Tokeshi, 1992). Such patterns are known to be affected by both methodological and biological factors (Brown, 1984; Collins & Glenn, 1997; Hui, 2012; McGeoch & Gaston, 2002; Mehranvar & Jackson, 2001; van Rensburg et al., 2000; Tokeshi, 1992). In the case of the former, survey parameters such as sampling procedure (e.g., sampling intensity, extent, and grain) may alter the SOFD patterns detected (Collins & Glenn, 1997; Hui, 2012; Jenkins, 2011; McGeoch & Gaston, 2002). For the latter, biotic factors and environmental conditions (e.g., habitat heterogeneity, productivity, disturbance, and study location) have also been shown to modify SOFD patterns (McGeoch & Gaston, 2002).

Overall, spatial variations in SOFD patterns have been more thoroughly investigated than temporal variation (Collins & Glenn, 1997; Korkeamäki et al., 2018; McGeoch & Gaston, 2002; Mehranvar & Jackson, 2001; Suhonen & Jokimäki, 2019), which has been analyzed in only a few studies. Most previous work has suggested that SOFDs tend to be rather stable over time (Collins & Glenn, 1997; Heino, 2008; Suhonen & Jokimäki, 2019), although seasonal variation in SOFD patterns has been reported in insects (Gaston & Lawton, 1989; Tokeshi, 1992).

There have been numerous published studies of SOFD patterns in animals and plants (Hui, 2012; Jenkins, 2011; McGeoch & Gaston, 2002), but thus far, there have not been any characterizations of SOFDs in freshwater macrophytes. Previous studies of plant assemblages have reported a wide variety of SOFD patterns (Hanski, 1982a; Hui, 2012; Jenkins, 2011; McGeoch & Gaston, 2002); due to this inconsistency, additional data on plant assemblages from different habitats are important to improve our understanding of SOFD variation in plant assemblages (Hanski, 1982a; Hui, 2012; Jenkins, 2011; McGeoch & Gaston, 2002). There seem to be some general rules for plant assemblage shape of SOFD patterns. Increasing study scale from local to regional shifted plant assemblage SOFD pattern from bimodal to unimodal satellite modal patterns (McGeoch & Gaston, 2002). Other factors which modify plant assemblage SOFD distribution patterns seem to be disturbance of habitat and succession of plant assemblages from unimodal satellite pattern to bimodal pattern to again unimodal satellite pattern (Jenkins, 2011).

As a study system, freshwater macrophytes are highly suitable for studies of SOFD patterns. Macrophyte species are well known, easy to identify, and frequently studied (see, e.g., Rintanen, 1996; Toivonen & Huttunen, 1995; and references therein). Moreover, there is the potential for a large degree of spatial and temporal variation among studies. In Finland, surveys have been conducted at scales ranging from local waterbodies to regional or national studies (Rintanen, 1996; Toivonen & Huttunen, 1995; Virola et al., 1999; Virola et al., 1999; Virolainen et al., 1999). In addition, several waterbodies have been resurveyed over a 40‐year period (Rintanen, 1996; Virola et al., 1999; Virolainen et al., 1999). The large amount of published data makes it possible to examine in detail how SOFD patterns in freshwater macrophyte assemblages can vary across different spatial and temporal scales.

In this study, SOFDs of macrophyte species assemblages in Finland were characterized, and changes in these patterns were evaluated across different spatial and temporal scales. For this, I analyzed previously published datasets on macrophyte occupancy frequencies in Finland (Rintanen, 1996; Toivonen & Huttunen, 1995; Virola et al., 1999; Virolainen et al., 1999) and assessed which of the main SOFD patterns (Collins & Glenn, 1997; Hui, 2012; Jenkins, 2011; McGeoch & Gaston, 2002; Mehranvar & Jackson, 2001; Tokeshi, 1992) best described macrophyte species occupancy in waterbodies. The five SOFD patterns used were (a) unimodal satellite dominant, (b) bimodal asymmetric, (c) bimodal symmetric, (d) bimodal truncated, and (e) linear (uniform). In the unimodal satellite‐dominant and bimodal truncated SOFD patterns, most species occupy only one or a few study sites (Brown, 1984; Hui, 2012; Jenkins, 2011; McGeoch & Gaston, 2002; Tokeshi, 1992). In contrast, in asymmetric and symmetric bimodal SOFD patterns, a higher number of species are found in all study sites (Hanski, 1982b; Hui, 2012; Jenkins, 2011; McGeoch & Gaston, 2002; Tokeshi, 1992).

The first aim of this study was to examine differences in SOFDs at two spatial scales: local and national. My prediction was that the number of core species would decrease and the number of satellite species would increase going from a local to a national scale due to increasing habitat heterogeneity and differences in species environmental and resource requirements (Collins & Glenn, 1997; McGeoch & Gaston, 2002). Second, I studied geographical variation in SOFD patterns in waterbodies between southern and central Finland. Geographical variations in the SOFD pattern may be due to variation in the regional species pool with different niche requirement in different location. Third, I investigated temporal variation in SOFD patterns over a period of 40 years. I expected that SOFD patterns would change due to the general eutrophication of waterbodies that has occurred in Finland over that time period (Rintanen, 1996) and anthropogenic disturbance around waterbodies (Hilli et al., 2007), which may increase or decrease number of macrophyte species. Finally, I tried to identify whether the observed macrophyte SOFD patterns are better explained by the dynamic metapopulation hypothesis (Hanski, 1982b) which is based on local extinction and colonization dynamics or the niche‐based hypothesis which is based on the idea that assemblages have many rare narrow‐niche specialist species and few common broad‐niche generalist species (Brown, 1984).

## 2 MATERIALS AND METHODS

### 2.1 Datasets

In this study, I used three previously published datasets. The first contained survey data collected at a national scale, specifically from 133 waterbodies along a 1,000‐km (60–69°N) latitudinal gradient in Finland. Each waterbody was surveyed twice, first in the 1930s and second in the 1980s. This dataset contained occupancy frequencies for 89 macrophyte species (Rintanen, 1996). The second dataset contained local data collected from 57 waterbodies in southern Finland, within a 25 × 25 km area near the cities of Tampere and Valkeakoski. Here, macrophyte species were surveyed between 1975 and 1979; this study included data on occupancy frequencies for 91 macrophyte species (Toivonen & Huttunen, 1995). The third dataset represented surveys of 25 waterbodies in central Finland (62°N, 26°E), in an area of 150 km^2^ near the villages of Konnevesi and Sumiainen. Each waterbody was investigated twice, first in the 1930s and again in 1996. This dataset contained occupancy frequencies for 48 macrophyte species (Virola et al., 1999; Virolainen et al., 1999). In all repeated macrophyte species survey were used the same methods as in previous more than 40 years apart. In both of the studies that surveyed communities at different time points (Rintanen, 1996; Virola et al., 1999; Virolainen et al., 1999), the same waterbodies were investigated using the same methods and survey intensity. The lakes were circled by boat, using a rake and a viewing box and partly by walking in the constant speed along the shores.

### 2.2 Statistical analyses

The methods used here were the same as in previous analyses of SOFD patterns (Hui, 2012; Jenkins, 2011), namely a multimodel inference approach based on ranked species occupancy curves (RSOCs) (Hui, 2012; Jenkins, 2011). First, I created binary (presence/absence) species‐by‐site matrices from each of the three datasets. Then, I calculated the occupancy frequency of each species as the sum of all the study sites in which it was found. Each macrophyte species' occupancy frequency was divided by the total number of waterbodies, and then, these relative occupancy values (*O_i_*) were sorted in decreasing order. Each macrophyte species had its own rank value, *R_i_*, which was inversely correlated with the relative occupancy value. For each dataset, I then performed five regression analyses in which the relative occupancy of a species (O_i_) was the dependent variable and *R_i_* was the independent variable. Finally, I determined which of the five core–satellite SOFD patterns gave the best fit for macrophyte species assemblages in waterbodies (Hui, 2012; Jenkins, 2011):

1. Unimodal satellite‐dominant SOFD pattern. RSOC: *O_i_* = *y*_0_ + *a* exp(−*bR_i_*) *y_0_*, *a*, *b* > 0 (exponential concave).
2. Bimodal symmetric SOFD pattern. RSOC: *O_i_* = *a*/(1 + exp(*bR*_i_ − *c*)), *a*, *b*, *c* > 0.14 (sigmoidal symmetric).
3. Bimodal asymmetric SOFD pattern. RSOC: *O_i_* = *a*[1 − exp(−*bR_i_* ^−^ *^c^*)], *a*, *b*, *c* > 0 (sigmoidal asymmetric).
4. Bimodal truncated SOFD pattern. RSOC: *O_i_* = *aR_i_^b^* exp(−*cR_i_*), *a*, *b*, *c* > 0 (power exponential).
5. Uniform SOFD pattern. RSOC: *O_i_* = *a* − *bR_i_*, *a*, *b* > 0 (linear).

In each case, *y*_0_, *a*, *b*, and *c* are estimated parameters.

As in previous studies (Hui, 2012; Jenkins, 2011), I calculated the nonlinear regressions using the Levenberg–Marquardt algorithm (upper limit on the number of iterations 999 iterations), and each parameter was estimated by means of ordinary least squares (OLS). I tried multiple initial guesses to ensure the resulting parameter estimates were the same and convergence occurred. I evaluated the assumptions of the regressions for normality of residuals, homogeneity of variance, and independent error terms, as well as the tails and shoulders of the data and models from different plotted graphs.

To compare the five different SOFD models, I used values of AICc (Akaike information criterion for small sample sizes); the smallest value indicates the best fit (Burnham & Anderson, 2000). If the difference between the values for two different models (ΔAICc = AICcmin − AICci) is higher than four, it is a strong indication of a better fit; ΔAICc values less than four indicate that the two models fit the data almost equally well (Anderson et al., 2000; Burnham & Anderson, 2000; Jenkins, 2011). There is some evidence that models for which ΔAICc values are less than seven can also be considered alternative models (Burnham et al., 2011).

Following the recommendations of McGeoch and Gaston (2002), all figures depict 10% occupancy classes and the number of macrophyte species in each. All analyses were performed using the IBM SPSS statistical package, version 26.

## 3 RESULTS

### 3.1 Species occupancy frequency

The average occupancy frequency for macrophyte species in Finland increased slightly from the 1930s to the 1980s, from 28.8 ± 28.6 (*SD*) (*n* = 82 species) to 32.9 ± 30.0 (*n* = 88) waterbodies, respectively. In other words, each species occupied about one‐fourth of all waterbodies (Table 1). In southern Finland, each species occurred in an average of 18 ± 15.6 (*n* = 91 species) waterbodies, meaning that each macrophyte species was found in about one‐third of waterbodies (Table 1). In central Finland, species occupancy frequencies were similar between the two time periods (11.6 ± 8.3, *n* = 41 species and 11.2 ± 8.7, *n* = 44 species, respectively), with each macrophyte species occupying almost half of the waterbodies (Table 1).

**TABLE 1:** Mean and *SD* of percent of waterbodies occupied by a given freshwater macrophyte species in different regions in Finland in different time periods

Region	Species	Mean	*SD*	Min	Max
Finland 1930s	82	25.5	25.3	0.9	96.5
Finland 1980s	88	29.1	26.6	0.9	96.5
Southern Finland	91	31.6	27.4	1.8	100.0
Central Finland 1934	41	46.3	33.0	4.0	100.0
Central Finland 1996	44	44.8	34.7	4.0	100.0

### 3.2 Assemblage SOFD patterns

**FIGURE 1:**
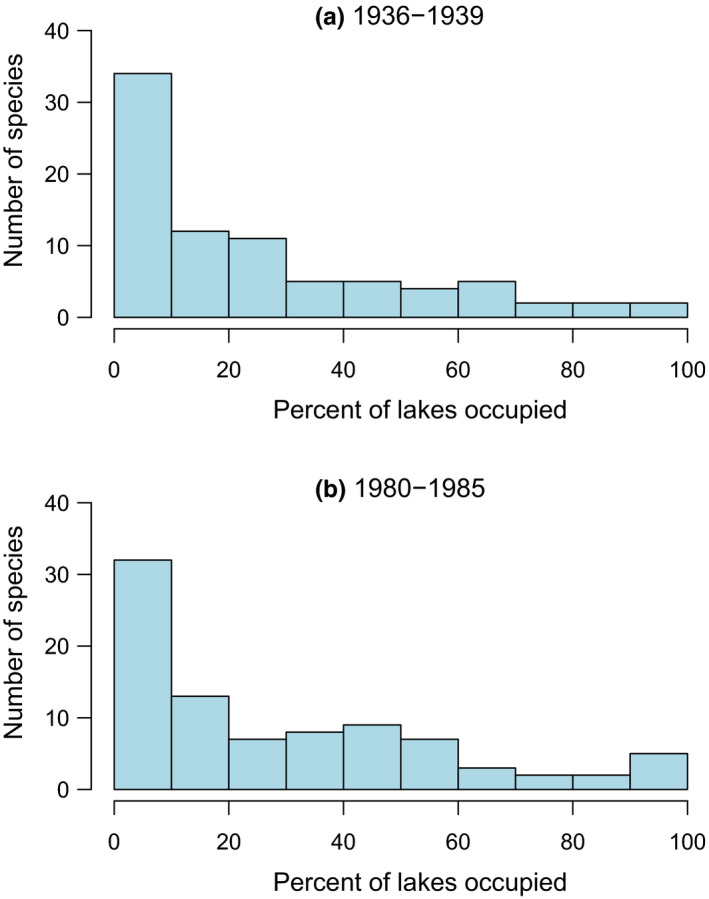
Species occupancy frequency distributions (SOFDs) showing the number of macrophyte species as a function of the proportion of waterbodies occupied (%) for 113 waterbodies. (a) 82 macrophyte species were surveyed in the 1930s, and (b) 88 macrophyte species were surveyed in the 1980s along a 1000‐km latitudinal gradient in Finland

**FIGURE 2:**
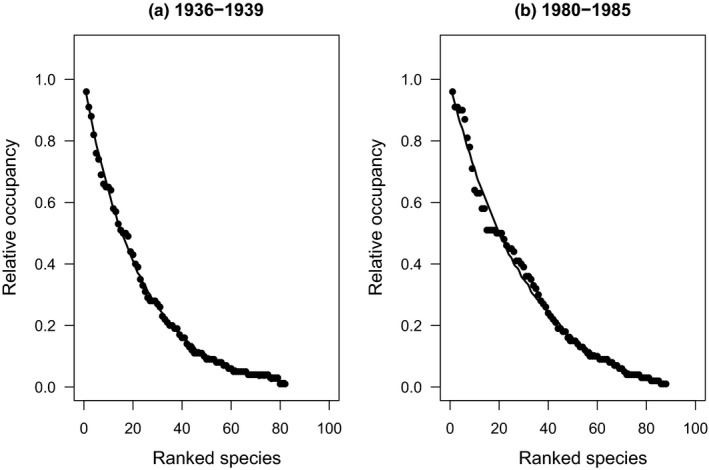
Observed ranked species occupancy curve (RSOC) for data of (a) years 1936–1939 and (b) years 1980–1985 in Finland. The continuous line based on predicted values on RSOC (exponential convex) models (a) *O_i_* = −0.013 + 1.002 * exp(−0.043 * *R_i_*) and (b) *O_i_* = −0.059 + 1.046 * exp(−0.031 * *R_i_*), where *O_i_* is predicted occupancy value of species *i* and *R_i_* is species *i* observed rank

**FIGURE 3:**
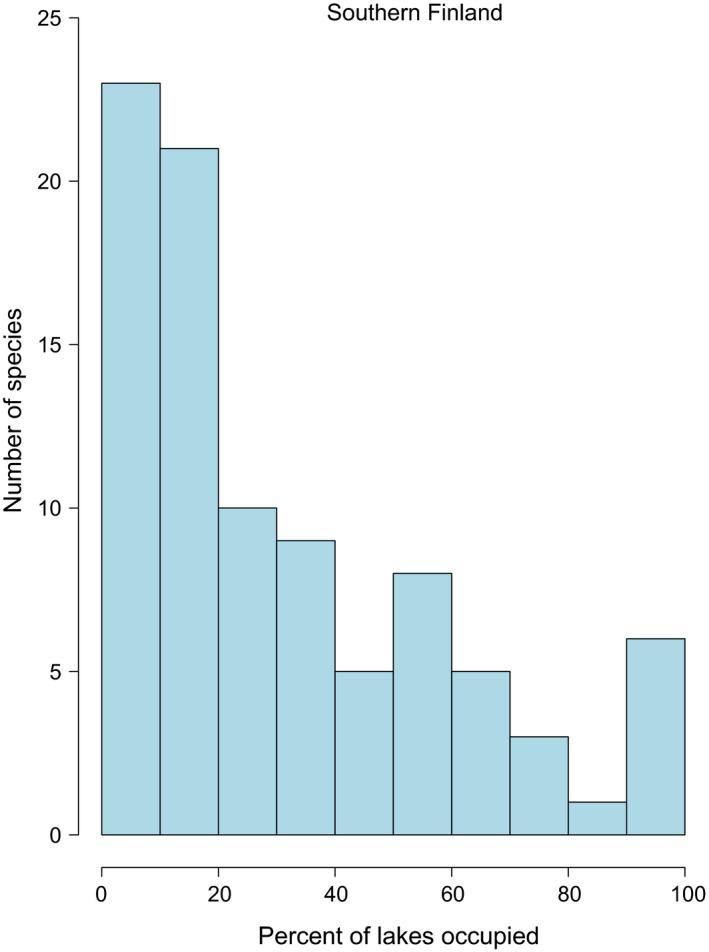
Species occupancy frequency distribution (SOFD) showing the number of macrophyte species (*n* = 91) as a function of the proportion of local waterbodies occupied (%) for 57 waterbodies in southern Finland

**FIGURE 4:**
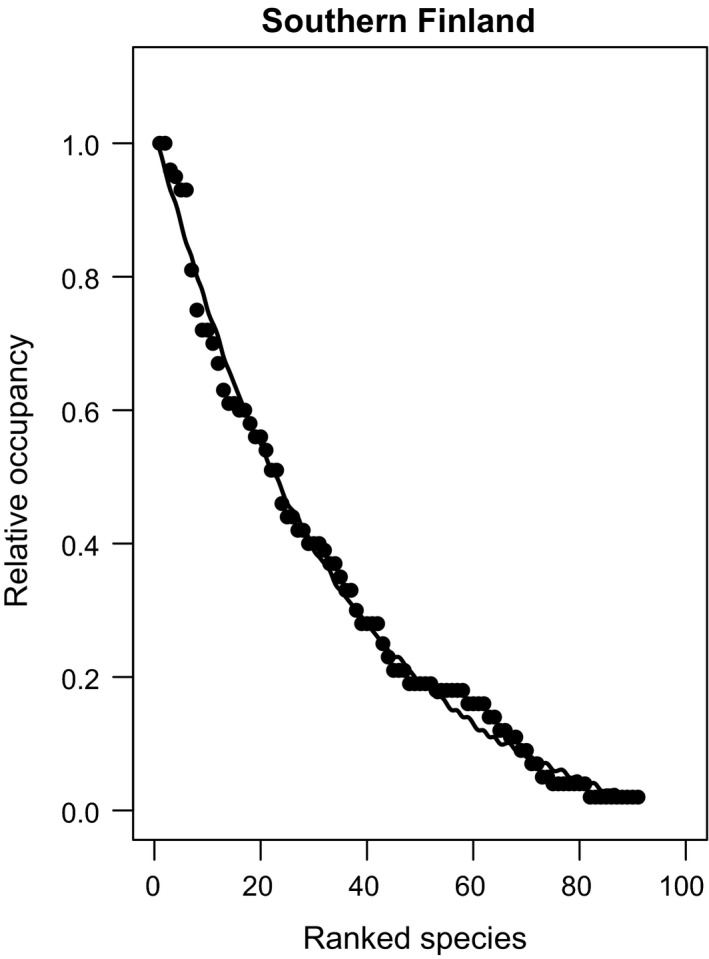
Observed ranked species occupancy curve (RSOC) for data of southern Finland. The continuous line based on predicted values on RSOC (exponential convex) models *O_i_* = −0.060 + 1.085 * exp(−0.029 * *R_i_*), where *O_i_* is predicted occupancy value of species *i* and *R_i_* is species *i* observed rank

**FIGURE 5:**
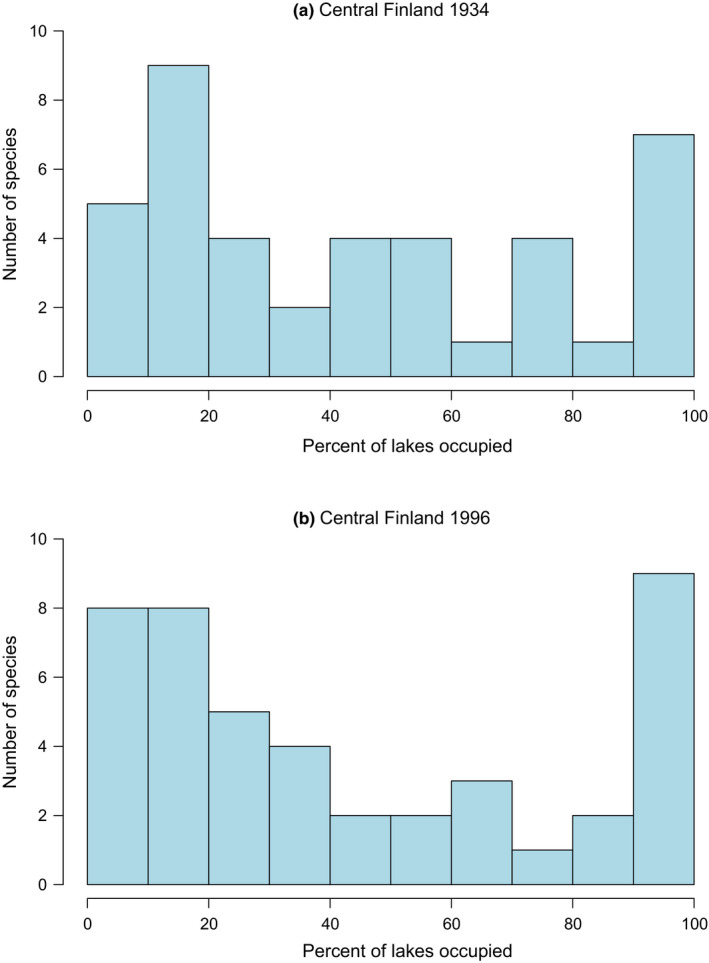
Species occupancy frequency distribution (SOFD) showing the number of macrophyte species as a function of the proportion of local waterbodies occupied (%) for 25 waterbodies in central Finland. (a) 41 macrophyte species in 1934 and (b) 44 macrophyte species in 1996

**FIGURE 6:**
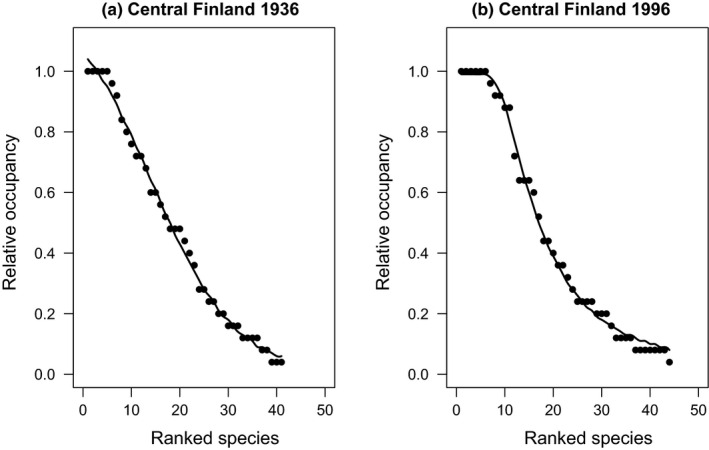
Observed ranked species occupancy curve (RSOC) for data of (a) years 1936 and (b) years 1996. The continuous line based on predicted values on RSOC models (a) *O_i_* = 1.27/(1 + exp(0.116 * *R_i_* − 1.645)) and (b) *O_i_* = 0.988 * [1 − exp(−356,996 * *R_i_* ^−2,196^)], where *O_i_* is predicted occupancy value of species *i* and *R_i_* is species *i* observed rank

I found both spatial and temporal variation in SOFD patterns (Table 2). For the large‐scale national dataset, representing 113 surveyed waterbodies, SOFDs followed a unimodal satellite pattern (Table 2; Figure 1 and Figure 2). The older national‐level data from the 1930s also fit equally well with a bimodal symmetric SOFD pattern (∆AICc 0.0; Table 2). At the local scale, there was geographical variation among studies (Table 2). Data from southern Finland followed a unimodal satellite SOFD pattern (Table 2; Figure 3 and Figure 4). Old data from central Finland, SOFD followed a bimodal symmetric pattern (Table 2; Figure 5a and Figure 6a). In central Finland, SOFD patterns seemed to shift from bimodal symmetric to bimodal asymmetric over the 40‐year time period examined (Table 2; Figure 5b and Figure 6b).

**TABLE 2:** Analysis of freshwater macrophyte species assemblages in waterbodies in Finland

Location	*n*	Model	Species	AICc	ΔAICc
Finland 1930s	113	**Unimodal satellite**	82	−705.2	0.0
		**Bimodal symmetric**	82	−705.2	0.0
		Bimodal asymmetric	82	−527.7	177.9
		Bimodal truncated	82	−699.1	5.7
		Uniform	82	−369.1	336.6
Finland 1980s	113	**Unimodal satellite**	88	−644.7	0.0
		Bimodal symmetric	88	−624.4	20.3
		Bimodal asymmetric	88	−510.6	134.2
		Bimodal truncated	88	−616.9	27.8
		Uniform	88	−421.7	223.0
Southern Finland	57	**Unimodal satellite**	91	−677.0	0.0
		Bimodal symmetric	91	−652.4	24.5
		Bimodal asymmetric	91	−528.0	149.0
		Bimodal truncated	91	−647.7	29.3
		Uniform	91	−441.0	236.0
Central Finland 1934	25	Unimodal satellite	41	−267.9	29.5
		**Bimodal symmetric**	41	−297.2	0.0
		Bimodal asymmetric	41	−243.3	53.9
		Bimodal truncated	41	−274.0	23.2
		Uniform	41	−230.8	66.4
Central Finland 1996	25	Unimodal satellite	44	−240.6	71.1
		Bimodal symmetric	44	−280.5	31.2
		**Bimodal asymmetric**	44	−311.7	0.0
		Bimodal truncated	44	290.3	21.4
		Uniform	44	−207.6	104.1

Note

“*n*” is the number of waterbodies surveyed. The five most common patterns of species occupancy frequency distribution (SOFD: unimodal satellite dominant, bimodal symmetrical, bimodal asymmetrical, bimodal truncated, and random) were assessed by fitting their associated ranked species occupancy curves (RSOCs) to each dataset. The AICc (Akaike information criterion for small sample sizes) as well as ΔAICc (=AICci − AICcmin) values are presented. The model with the lowest AICc was considered the best of the tested models, and alternative models with ΔAIC smaller than seven were considered equally valid (Burnham et al., 2011). The most fitted model is in bold.

## 4 DISCUSSION

This study examined SOFD patterns for macrophyte species in Finland at different spatial and temporal scales. On average, each macrophyte species occupied at least one‐fourth of waterbodies surveyed, and the average frequency was higher in central than southern Finland.

As predicted, I found that SOFD patterns shifted across different spatial scales: Going from a local to a national scale, the number of core species decreased and the number of satellite species increased. With respect to temporal differences, however, I found only minor changes in SOFD patterns.

### 4.1 Species occupancy frequency

I found that, on average, each macrophyte species occupied at least one‐fourth of waterbodies, with higher average frequencies in central than southern Finland. Similarly, a study of odonates in Fennoscandia reported that relative occupancy frequency increased at higher latitudes (Korkeamäki et al., 2018). The decrease in macrophyte occupancy frequency at southern sites may be due to latitudinal variation in the size of the species pool. A previous study found that the species pool of freshwater macrophytes in Finland varied at different latitudes, and species richness decreased going from the south to the north (Heino & Toivonen, 2008). In odonates, a decrease in species richness in waterbodies was found to be associated with an increase in the species occupancy frequency (Korkeamäki et al., 2018).

### 4.2 Assemblage SOFD patterns

Both McGeoch and Gaston (2002) and Hui (2012) estimated that one‐fourth of SOFD patterns were bimodal symmetric. The same frequency was found in this study, in which this type of SOFD was found in older surveys (1930s) of freshwater macrophyte assemblages at the local scale in central Finland and at the national scale. However, in the study of Hui (2012, Appendix S1), the two most frequent SOFD patterns in plants were bimodal truncated and unimodal satellite dominant of which were also found here.

### 4.3 National scale

At the national scale, macrophyte species assemblages appeared to follow a unimodal satellite‐dominant SOFD pattern. As expected, the number of core species decreased and the number of satellite species increased with a shift from the local to the national scale. The observed SOFD pattern gave support to the niche‐based hypothesis, which is based on the idea that assemblages have many rare narrow‐niche specialist species and few common broad‐niche generalist species (Brown, 1984). The national scale data were collected along large 1,000‐km (60–69°N) latitudinal gradient in Finland. In so wide extent scale, the environment variables will come more different and all observed macrophyte species niche requirements will be met less frequently (Toivonen & Huttunen, 1995). This will decrease the number of species in the core class and increase species in the satellite class (Brown, 1984; McGeoch & Gaston, 2002). As discussed above in the context of species occupancy frequencies, this pattern could also be the result of variation in the size of the macrophyte species pool in different locations in Finland (Heino & Toivonen, 2008). Such changes in species richness and assemblage position typically increase the number of satellite species and decrease the number of core species. Overall, the changes in SOFDs observed here for freshwater macrophyte assemblages are consistent with most previously published studies that have assessed the effects of increasing study scale (extent) (Brown, 1984; Collins & Glenn, 1997; Korkeamäki et al., 2018; McGeoch & Gaston, 2002).

### 4.4 Local scale

At a local scale, clear differences were observed between the SOFD patterns of freshwater macrophyte species in southern and central Finland. In southern Finland, freshwater macrophyte assemblages followed a unimodal satellite‐Tdominant pattern, whereas in central Finland, these assemblages demonstrated a bimodal symmetric (in early surveys) or bimodal asymmetric (in later surveys) pattern. A similar spatial pattern was reported in damselfly and dragonfly assemblages in Fennoscandia (Korkeamäki et al., 2018). For both groups of study organisms, it seems likely that the richer species pool in the southern locations skewed the unimodal SOFD pattern into a bimodal one. Moreover, at local scale the environment is autocorrelated due to similar climate and environmental conditions and numerous species met their similar niche requirements, which increase species in the core class. This may explain differences in SOFD patterns between local and the national scales.

### 4.5 Temporal variation

This study detected only a small temporal variation in the SOFD patterns of freshwater macrophyte assemblages. Stability in SOFD pattern may be due to relative stable environmental conditions. Moreover, the species local extinctions and colonization seem to be in balance. So, most of species survived between two survey periods and relative few species vanished and few new species arrived to the waterbodies (Virola et al., 1999). This small shift in species occupancy frequencies may be due to eutrophication and changes in land use around waterbody (Hilli et al., 2007; Lindholm et al., 2021; Rintanen, 1996). Similarly, previous studies have suggested that SOFDs tend to be fairly stable over time (Collins & Glenn, 1997; Heino, 2008; Suhonen & Jokimäki, 2019). Although the area of waterbodies (grain size) did vary within the study, this did not affect the observed SOFD patterns because the same methods were used in both surveys (more than 40 years apart). In both of the studies that surveyed assemblages at different time points (Rintanen, 1996; Virola et al., 1999; Virolainen et al., 1999), the same waterbodies were investigated using the same methods and survey intensity, which should minimize the effect of possible sampling artifacts (McGeoch & Gaston, 2002). The most plausible explanation for the changes in species occupancy frequencies is likely eutrophication, other change in water quality, and changes in the landscape surrounding waterbodies that have occurred over time (Hilli et al., 2007; Lindholm et al., 2021; Rintanen, 1996).

### 4.6 Detectability of species

In this study, I used “presence–absence” data to determine occupancy. However, the issue of species classified as absent when they are in fact present but not detected is an important one for studies of SOFD patterns. The detectability may be higher problem in moving animals than sessile plants such as aquatic macrophytes, but imperfect detection of plants may be also common (Chen et al., 2013). The probability a given species is present when it is not detected may modify shape of SOFD pattern. It seems more likely that common species detected more often than rare species due to investigator higher searching image for common and most abundant species. However, rare and less abundant species were more often missed due to low detectability. If sampled study sites had several rare species, which were not detected, then satellite species frequencies were lower than real assemblage. For example, observed bimodal symmetric pattern would in reality be bimodal asymmetric or even unimodal satellite‐dominant SOFD pattern. Scientists try to minimize the probability of missing a species by visiting sites multiple times and then estimate species detection probabilities (MacKenzie et al., 2006). However, this is not possible for old and already published datasets, which are interesting for long‐term monitoring programs and suitable to test several ecological hypotheses. One solution may be simulation data with adding or removing species from original data and test how stable are results (see also Chen et al., 2013). For example, a bootstrapping method would determine whether the results generated from this research is stable or not. This idea require more investigation in the future.

### 4.7 Multiple alternative models

One fundamental issue in studies based on snapshots of quasistationary distributions such as SOFD patterns is that several alternative models based on substantially different or even contradictory biological assumptions may predict the same or very similar form of stationary distribution. While I am not aware of any attempt to derive the RSOC equations employed in this study using different biological assumptions, I found that the AIC‐based model selection procedure was not able to distinguish between unimodal satellite‐dominant and bimodal symmetric macrophyte SOFD patterns in data from the 1930s (Table 2).

In this case, the Akaike information criterion gave equal support to two alternative ecological hypotheses: the ecological niche‐based hypothesis (Brown, 1984), which predicts unimodal satellite‐dominant SOFD pattern, and the dynamic metapopulation hypothesis (Hanski, 1982b), which predicts the bimodal symmetric SOFD pattern. So, the Akaike information criterion gave equal support to alternative models derived from rather different biological assumptions, both of which were able to predict the observed distribution pattern of aquatic macrophyte assemblages well. It appears, then, that the models are mainly useful in providing alternative parametric regression equations that can be used to objectively classify the shapes of observed SOFD and RSOC patterns. When used in this manner, with parameter values being estimated by a statistical fitting procedure instead of by direct measurement, models that fit the data well should not be assumed to provide conclusive support for the biological assumptions used to derive them, as it may well be possible to derive the same or similar regression equations using markedly different biological assumptions.

### 4.8 Conclusions

To conclude, at a national scale, the results of this study demonstrate that freshwater macrophyte assemblages follow a unimodal satellite dominant SOFD pattern, with many rare species and few common ones. This observed SOFD pattern seems to be due to wide extent scale, the environment variables will come more different and all observed macrophyte species niche requirements will be met less frequently (Toivonen & Huttunen, 1995). At the local scale, the results varied across both space and time probably due to other change in water quality, eutrophication, and changes in the landscape surrounding waterbodies (Hilli et al., 2007; Lindholm et al., 2021; Rintanen, 1996). The highest degree of support was found for the bimodal symmetric, unimodal satellite‐dominant, and bimodal truncated SOFD patterns. The relative representation of core and satellite species in assemblage composition and occupancy frequencies varied among waterbodies, probably due to differences in the species pool size. The general pattern detected here for macrophyte assemblages—numerous rare species and only a few common ones—is similar to what several previous studies have reported for macroinvertebrates in aquatic environments (Heino, 2008, 2015; Jenkins, 2011; Korkeamäki et al., 2018; Renner et al., 2020; Tokeshi, 1992; Verberk et al., 2010). These findings provide slightly more support for the niche‐based model of community assembly (Brown, 1984) than the dynamic metapopulation model (Hanski, 1982b), because the majority of macrophyte assemblages had unimodal distributions with a large number of satellite species. By contrast, support for the dynamic metapopulation model seems to be slightly stronger in terrestrial plant assemblages (Collins & Glenn, 1997; Hanski, 1982a). Together with previous work on odonate assemblages in northern Europe (Korkeamäki et al., 2018), the present study serves as a useful starting point, but more studies are required from different locations in order to help us more fully understand the factors underlying geographical variation in SOFD patterns.

## CONFLICT OF INTEREST

No conflict of interest.

## AUTHOR CONTRIBUTION

**Jukka Suhonen:** Conceptualization (equal); Data curation (equal); Formal analysis (equal); Methodology (equal); Visualization (equal); Writing‐original draft (equal); Writing‐review & editing (equal).

## Data Availability

In this study were used only published data.
